# Dutch women’s intended participation in a risk-based breast cancer screening and prevention programme: a survey study identifying preferences, facilitators and barriers

**DOI:** 10.1186/s12885-020-07464-2

**Published:** 2020-10-06

**Authors:** Linda Rainey, Daniëlle van der Waal, Mireille J. M. Broeders

**Affiliations:** 1 Radboud Institute for Health Sciences, Radboud University Medical Center, PO Box 9101, 6500 HB Nijmegen, The Netherlands; 2 Dutch Expert Centre for Screening, PO Box 6873, 6503 GJ Nijmegen, The Netherlands

**Keywords:** Breast cancer, Risk assessment, Screening, Acceptability, Prevention

## Abstract

**Background:**

Risk-based breast cancer screening may improve the benefit-harm ratio of screening by tailoring policy to a woman’s personal breast cancer risk. This study aims to explore Dutch women’s preferences regarding the organisation and implementation of a risk-based breast cancer screening and prevention programme, identifying potential barriers and facilitators to uptake.

**Methods:**

A total of 5110 participants in the Dutch Personalised RISk-based MAmmography screening (PRISMA) study were invited, of whom 942 completed a two-part web-based survey. The first part contained questions about personal characteristics; for the second part, women were randomly assigned to one of four hypothetical breast cancer risk scenarios (i.e. low, average, moderate, or high) with subsequent tailored screening and prevention advice. Descriptive statistics are used to present women’s organisational preferences. Univariable and multivariable logistic regression analyses were performed using seven proxy measures for acceptability of risk-based screening (e.g., interest in risk) and risk-based prevention (e.g., willingness to change diet).

**Results:**

Interest in breast cancer risk was high (80.3%). Higher assigned risk scenario was most consistently associated with acceptance of tailored screening and prevention recommendations. Increased acceptance of lifestyle changes was additionally associated with higher education. Having a first degree family history of breast cancer decreased women’s motivation to participate in preventative lifestyle measures. Acceptability of medication was associated with a woman’s general beliefs about the (over)use and benefit-harm balance of medication.

**Conclusions:**

Dutch women generally appear in favour of receiving their breast cancer risk estimate with subsequent tailored screening and prevention recommendations. However, women’s level of acceptance depends on their assigned risk category. Offering tailored screening and prevention recommendations to low-risk women will be most challenging. Educating women on the benefits and harms of all risk-based screening and prevention strategies is key to acceptability and informed decision-making.

## Background

Risk-based breast cancer screening may improve the benefit-harm ratio of screening by tailoring policy to a woman’s personal breast cancer risk [1, 2]. This could, for example, entail less intensive screening for women at lower risk and more intensive screening for women at higher risk, e.g., offering higher risk women supplemental screening with different modalities, such as MRI or ultrasound. Subsequent risk-tailored screening policy could also potentially correspond more closely to a woman’s individual preferences. Informing women about their breast cancer risk also provides opportunities to educate them on breast cancer prevention [3], providing tailored advice on healthy lifestyle behaviours and risk-reducing medication.

Assessing the breast cancer risk of a population sample of women eligible for screening has been shown to be feasible using a modified version of the Tyrer-Cuzick breast cancer risk prediction model, including information on breast density and genetics [4]. Moreover, women in the United Kingdom have expressed high interest in knowing their breast cancer risk [5, 6]. It is unclear whether Dutch women are equally open to having their breast cancer risk assessed and participating in tailored screening strategies based on this personal breast cancer risk. Women in the Netherlands receive an invitation for a screening mammogram every 2 years between the ages of 50–75 years. The Personalised RISk-based MAmmography screening (PRISMA) study is a large prospective cohort study which is currently being performed in the setting of the Dutch national screening programme. The aim of the PRISMA study is to update and validate an existing breast cancer risk prediction model to guide screening policy in the Netherlands by collecting extensive information on breast cancer risk factors. Although the optimal risk-based screening strategies have not yet been established, certain scenarios may include less frequent screening of low-risk women for a shorter number of years, whereas high-risk women could receive more frequent and prolonged screening, potentially with supplemental imaging techniques. Reduced screening intensity in particular may be less acceptable in the setting of an established screening programme [7]. Women in the Netherlands have been informed that the benefits of biennial mammography screening outweigh the harms and any changes might be met with scepticism.

A first qualitative exploration of the acceptability of risk-based breast cancer screening and prevention among a small group of women eligible for screening in the Netherlands, the United Kingdom, and Sweden confirmed the controversial nature of reduced screening intensity [8]. Women also expressed reluctance about participating in preventative practices to decrease their breast cancer risk. It is important that these concerns are studied further in a larger group of women, since they may affect potential future implementation of the programme. Additionally, by exploring women’s preferences regarding the organisation of risk-based screening and prevention, we ensure that it optimally reflects women’s needs, facilitating potential uptake in the future. Therefore, the present survey study aims to explore eligible Dutch women’s perceptions and preferences regarding the organisation and implementation of a risk-based breast cancer screening and prevention programme. Moreover, we aim to explore whether any of women’s personal characteristics are associated with acceptability of different risk-based screening and prevention approaches. This will enable us to further identify any potential barriers and facilitators to uptake.

## Methods

### Design

Cross-sectional data were collected between June and October 2018 in the Netherlands using a web-based survey which was designed using qualitative focus group data [8]. Ethics approval was acquired from the regional ethics committee CMO Arnhem-Nijmegen (2015–1773). Informed consent was obtained online prior to the start of the survey.

### Participants

Women were selected from the participant database of the PRISMA study. PRISMA participants who met the Dutch screening eligibility criteria at the time of the survey study, i.e. aged 50–75 years and without a breast cancer diagnosis, and who consented to being approached for follow-up studies were invited to take part in this survey study. All participants were unaware of their breast cancer risk at time of participation in the survey study. We invited 5110 women in the Netherlands from three screening regions covering the North, East, and South-West of the country by sending an e-mail containing the survey’s participant information sheet. Women received a second email with a link to the online survey after they professed interest in participating via telephone or email.

### Procedure

**Fig. 1:**
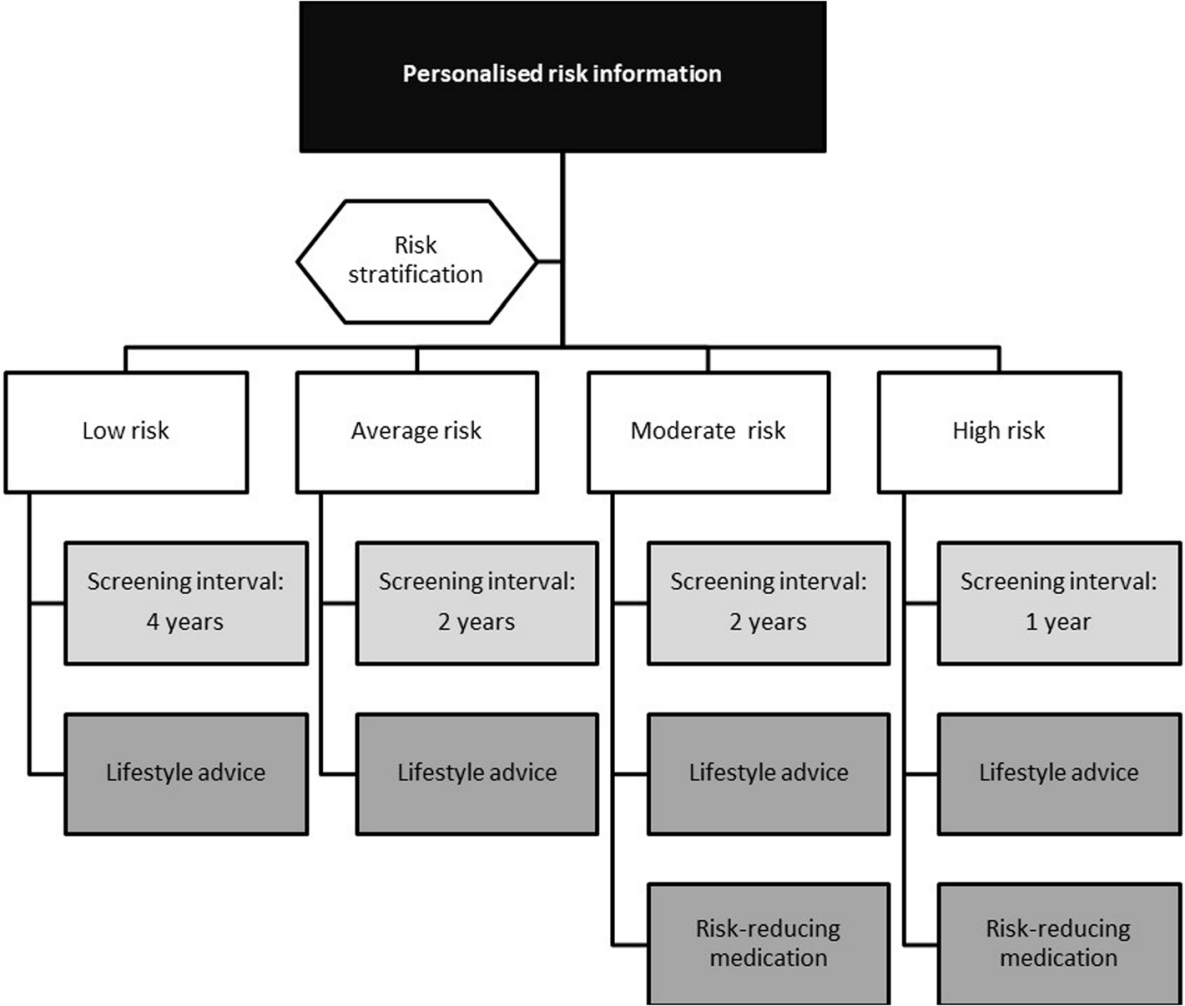
Hypothetical breast cancer risk categories with subsequent screening and prevention advice

The survey took 20–30 min to complete and contained two parts. In the first part, all participants answered questions about different aspects of their lives, e.g., demographics, family history, and general health. For the second part, we used a computer-generated randomisation scheme to randomly assign participants to one of four hypothetical breast cancer risk scenarios, i.e., low, average, moderate, or high, with subsequent screening and prevention recommendations (Fig. 1). Each scenario contained a tailored screening interval, i.e. 4 years for low risk, 2 years for average and moderate risk, and 1 year for the high risk scenario. To explore acceptability, women were asked to indicate perceived need for (1) supplemental mammography screening outside of the national screening programme and (2) increased breast self-examination based on this personalised screening interval. Women who completed the low or high risk scenarios were additionally asked to indicate their preferred screening interval and starting age. All four risk scenarios provided women with the option of improving their diet, exercise and alcohol consumption habits to decrease their hypothetical breast cancer risk. The moderate and high risk scenarios also provided women with the option of taking oral tamoxifen for 5 years to reduce their hypothetical risk. A copy of the survey is available upon request.

### Measures

#### Outcome variables

Acceptability of risk-based screening was assessed using three proxy measures. These measures are based on results from a previous focus group study [8] which indicated that women who were accepting of risk-based screening and prevention showed (1) interest in knowing their breast cancer risk (yes vs. no or do not know), (2) had a low perceived need for supplemental mammography screening outside of the national screening programme (yes vs. no or do not know), and (3) a low perceived need for breast self-examination (yes vs. no or do not know). Acceptability of risk-based prevention was assessed using four proxy measures: willingness to change diet (yes vs. no or do not know), become more physically active (yes vs. no or do not know), limit alcohol intake (yes vs. no or do not know), and willingness to consider risk-reducing medication for the hypothetical moderate and high risk groups (yes vs. no or do not know).

#### Determinants

##### Sociodemographic variables

Data on participants’ age (continuous), educational attainment (lower education, higher secondary education, higher vocational education including university), marital status (living together versus alone), and body mass index (BMI in kg/m^2^, continuous) were acquired.

##### Medical history

We included information on women’s medical history, i.e. family history of breast cancer (yes/no), personal history of benign breast disease (yes/no), diagnosed with one of the following medical conditions: cardiovascular disease, stroke, high blood pressure, asthma, chronic bronchitis, COPD, diabetes, ulcer, kidney disease, liver disease, anaemia, thyroid disease, depression, arthritis, and backache (< 2, ≥ 2 to indicate co-morbidity), and current medication use for one or more of these conditions (yes/no).

##### General health

General health on the day of participation was measured with the general EuroQol visual analogue scale (EQ VAS) [9]. It records self-rated health on a vertical scale ranging from 0 (worst imaginable health state) to 100 (best imaginable health state). It was included in analyses as a continuous measure.

##### Health anxiety

Health anxiety was measured with the validated 14-item Short Health Anxiety Inventory [10, 11]. Each item contains four statements. Participants are asked to choose the statement that best describes their feelings from the past 6 months on a scale from 0 (low health anxiety) to 3 (high health anxiety). A total sum score was calculated which was used as a continuous measure in analyses.

##### Health locus of control

Health locus of control refers to a person’s beliefs or expectations about which persons or other factors determine their health [12]. It was measured with the widely-used and validated 18-item Multidimensional Health Locus of Control Scales [12, 13]. There are three scales of each six items assessing an internal locus of control, a powerful others locus of control, and a chance locus of control. Answers are provided on a 6-point Likert scale ranging from strongly disagree (1) to strongly agree (6). For each scale, a total score can be calculated by summing the items. For analyses we used the highest scale score for each participant to indicate their main locus of control, resulting in a variable with four categories, i.e. 1 ‘mostly internal’, 2 ‘mostly powerful others, e.g., physicians’, 3 ‘mostly chance’, and 4 ‘no clear preference’.

##### Life events

Life events experienced in the past year were measured using an abbreviated version of the validated Holmes-Rahe Stress Inventory [14]. Based on our previously described focus group results [8], we included 10 life events that were considered relevant to the potential adoption of risk-based breast cancer screening and prevention. For analyses, we used a cut-off score of < 2 and ≥ 2 life events in the past year to enable meaningful group analysis.

##### Beliefs about medicines

General beliefs about medicines were measured with the validated 8-item Beliefs About Medicines Questionnaire [15]. It comprises two 4-item scales: the first scale assesses the belief that medicines are harmful, addictive, poisonous and should not be taken continuously; the second scale assesses the belief that medicines are overused by physicians. Answers are scored on a 5-point Likert scale ranging from 1 (strongly disagree) to 5 (strongly agree). Higher scores indicate a stronger belief. A sum score was calculated for both subscales and used continuously in analyses.

##### Perceived breast cancer risk

Perceived breast cancer risk was assessed using the item ‘How do you perceive your own risk of developing breast cancer in comparison with a woman of your age?’, with five response options: low, below average, average, above average, and high. For analyses we distinguished between below average (low and below average), average, and above average (above average and high) due to limited subgroup sample sizes.

### Statistical analyses

Descriptive statistics were presented to establish women’s general characteristics and preferences regarding risk-based screening and prevention. Women’s characteristics and preferences were further stratified by hypothetical risk scenario (i.e. low, average, moderate, and high). Next, we performed exploratory univariable and multivariable regression analyses to assess associations between the determinants and the seven outcome measures which function as proxies for women’s acceptability of risk-based screening and prevention. The selected associations that were explored were based on literature and our previous focus group study [8]. In the multivariable analyses, all associations were corrected for potential confounders. Analyses were performed with IBM SPSS version 22 (Armonk, NY: IBM Corp).

## Results

### Participant characteristics

A total of 942 women completed the survey. (response rate 18.4%). At the start of the survey women were randomly assigned to either the hypothetical low risk (*n* = 236), average risk (*n* = 228), moderate risk (*n* = 239), or high risk scenario (*n* = 239). Table 1 provides an overview of the general characteristics of all participants and stratified by risk scenario. Women’s characteristics do not differ based on hypothetical risk. Women were on average 59 years of age (SD = 6.3), moderately (27.9%) to highly (45.2%) educated and living with a partner (77.3%). Although, on average, women rated their health positively with a score of 82.1 (SD = 14.2), 45% had 2 or more medically diagnosed conditions and 41.5% was taking medication at time of participation. Over one-fifth (21.7%) of the participants had a first-degree relative with breast cancer. Most women estimated their personal breast cancer risk to be lower than average (39.8%) to average (53.8%). Women generally had either an internal (32.4%) health locus of control or felt that health was mostly due to chance (48.2%).

**Table 1:** General characteristics of the participants (*n* = 942)

	All women ***N*** = 942		Low risk ***N*** = 236		Average risk ***N*** = 228		Moderate risk ***N*** = 239		High risk ***N*** = 239	
Age (years), mean (SD)	59.0	(6.3)	58.7	(6.3)	58.6	(6.5)	58.9	(5.9)	59.8	(6.3)
Education level, n (%)										
Lower education	249	(26.4)	65	(27.6)	58	(25.4)	63	(26.4)	63	(26.4)
Higher secondary education	372	(39.5)	82	(34.7)	97	(42.5)	96	(40.2)	97	(40.6)
Higher vocational qualification	321	(34.1)	89	(37.7)	73	(32.1)	80	(33.4)	79	(33.0)
Marital status, n living with partner (%)	729	(77.3)	187	(79.2)	180	(78.9)	188	(78.7)	174	(72.8)
First-degree family history breast cancer, n yes (%)	204	(21.7)	47	(19.9)	52	(22.8)	47	(19.7)	58	(24.3)
Body mass index (kg/m^2^), mean (SD)^a^	26.3	(4.8)	26.5	(4.9)	26.1	(4.6)	26.6	(5.5)	26.0	(4.1)
Medical condition, n ≥ 2 diagnosed (%)^b^	424	(45.0)	119	(50.4)	96	(42.1)	103	(43.1)	106	(44.4)
Current medication use, n yes (%)	391	(41.5)	109	(46.2)	85	(37.3)	91	(38.1)	106	(44.4)
Current MHT^c^ use, n yes (%)	33	(3.5)	9	(3.8)	5	(2.2)	12	(5.0)	7	(3.0)
Benign breast disease, n yes (%)	272	(28.9)	79	(33.5)	64	(28.1)	68	(28.5)	61	(25.5)
Perceived breast cancer risk (%)										
Low	182	(19.3)	48	(20.3)	44	(19.3)	47	(19.7)	43	(18.0)
Below average	186	(19.7)	45	(19.1)	49	(21.5)	40	(16.7)	52	(21.8)
Average	498	(52.9)	126	(53.4)	121	(53.1)	130	(54.4)	121	(50.6)
Moderate	54	(5.7)	10	(4.2)	10	(4.4)	16	(6.7)	18	(7.5)
High	5	(0.5)	2	(0.8)	1	(0.4)	1	(0.4)	1	(0.4)
Missing value	17	(1.8)	5	(2.1)	3	(1.3)	5	(2.1)	4	(1.7)
General health score, mean (SD)	82.1	(14.2)	81.3	(14.4)	81.7	(15.5)	83.8	(12.8)	81.5	(13.9)
Life events, *n* ≥ 2 (%)^d^	264	(28.0)	74	(31.4)	64	(28.1)	63	(26.4)	63	(26.4)
HLOC^e^, n (%)										
Internal	300	(31.8)	78	(33.1)	69	(30.3)	73	(30.5)	80	(33.5)
Physician	67	(7.1)	11	(4.7)	11	(4.8)	27	(11.3)	18	(7.5)
Chance	447	(47.5)	115	(48.7)	111	(48.7)	107	(44.8)	114	(47.7)
No clear preference	113	(12.0)	29	(12.3)	34	(14.9)	27	(11.3)	23	(9.6)
Missing value	15	(1.6)	3	(1.3)	3	(1.3)	5	(2.1)	4	(1.7)
Belief in medicines, mean (SD)										
Harm	9.0	(2.5)	9.0	(2.5)	9.2	(26.7)	9.0	(2.5)	9.0	(2.5)
Overuse	11.5	(3.3)	11.6	(3.3)	11.6	(3.4)	11.6	(3.3)	11.3	(3.3)
Health anxiety, mean (SD)	7.7	(4.0)	7.6	(4.1)	7.7	(3.8)	7.6	(3.8)	7.8	(4.2)

^a^7 women with missing value (0.7%); ^b^ ≥ 2 medical diagnosis to indicate co-morbidity; ^c^ MHT = menopausal hormone therapy; ^d^ ≥ 2 life events to enable meaningful group comparisons; ^e^ HLOC = health locus of control

### Risk-based screening and prevention preferences

Table 2 describes women’s preferences regarding different aspects of a potential risk-based breast cancer screening and prevention programme. Women generally wanted to know their breast cancer risk (80.3%), whereas 10.5% of women was unsure. Most women were prepared to have a mammogram (96.2%), complete a questionnaire (95.9%), and provide a blood sample (97.6%) for the risk assessment. Although most women who completed the low risk scenario found it acceptable to receive their risk result in a letter (86.1%), this was not the case for other risk groups, with, for example, only around 50% of high-risk women finding this acceptable. The perceived need for a risk consultation also increased with risk, with 56.7% of low-risk women expressing this need compared with 70.2%, 91.5% and 93.2% of average, moderate, and high risk women. Women generally preferred a face-to-face (69.4%) or telephone (14.5%) risk consultation from either a GP (57.2%) or an oncologist (31.1%). Few women (3.5%) were interested in videoconferencing possibilities. More than 75% of women would use a website with additional information about risk-based screening and prevention.

**Table 2:** Women’s preferences regarding risk-based screening and prevention for each hypothetical risk scenario

	All women ***N*** = 942		Low risk ***N*** = 236		Average risk ***N*** = 228		Moderate risk ***N*** = 239		High risk ***N*** = 239	
Want to know breast cancer risk, n (%)^a^										
Yes	756	(80.3)								
No	70	(7.4)								
Don’t know	99	(10.5)								
Missing value	17	(1.8)								
Willing to get mammogram, n (%)	727	(96.2)								
Willing to complete questionnaire, n (%)	725	(95.9)								
Willing to provide blood sample, n (%)	738	(97.6)								
Risk result in letter, n acceptable (%)^a^	621	(67.1)	199	(86.1)	160	(70.2)	144	(61.5)	118	(50.2)
Need for consultation, n yes (%)^a^	722	(76.6)	131	(56.7)	158	(70.2)	214	(91.5)	219	(93.2)
Preferred risk counsellor, n (%)^b^										
General practitioner	539	(57.2)	91	(38.6)	126	(55.3)	169	(70.7)	153	(64.0)
Oncologist	293	(31.1)	38	(16.1)	58	(25.4)	94	(39.3)	103	(43.1)
Nurse	93	(9.9)	21	(8.9)	24	(10.5)	24	(10.0)	24	(10.0)
Geneticist	77	(8.2)	14	(5.9)	10	(4.4)	29	(12.1)	24	(10.0)
Radiologist	64	(6.8)	11	(4.7)	15	(6.6)	24	(10.0)	14	(5.9)
Radiographer	63	(6.7)	15	(6.4)	18	(7.9)	19	(7.9)	11	(4.6)
Use website, n yes (%)^c^	730	(79.0)	167	(72.6)	172	(76.4)	203	(86.8)	188	(80.0)
Supplemental screening, n yes (%)^c^	370	(39.3)	63^d^	(27.4)	89^e^	(39.6)	117^e^	(50.0)	101^f^	(43.0)
Preferred screening interval low risk										
2-year			116	(50.7)						
3-year			30	(13.1)						
4-year			30	(13.1)						
5-year			13	(5.7)						
Don’t know			18	(7.8)						
Other			22	(9.6)						
Preferred screening start age low risk										
50 years			64	(27.9)						
51 years			3	(1.3)						
52 years			12	(5.2)						
53 years			8	(3.5)						
54 years			7	(3.1)						
55 years			37	(16.2)						
56 years			3	(1.3)						
57 years			3	(1.3)						
58 years			3	(1.3)						
59 years			1	(0.4)						
60 years			13	(5.7)						
Other			43	(18.8)						
Don’t know			32	(14.0)						
Preferred screening interval high risk										
6-month									31	(30.7)
1-year									51	(50.5)
18-month									1	(1.0)
2-year									4	(4.0)
Don’t know									6	(5.9)
Other									8	(7.9)
Preferred screening start age high risk										
40 years									60	(59.4)
42 years									1	(1.0)
45 years									5	(5.0)
50 years									2	(2.0)
Other									22	(21.8)
Don’t know									11	(10.9)
Increased breast self-exam (% yes)^g^	597	(64.7)	65	(28.4)	132	(58.7%)	204	(87.2)	196	(83.4)
Change diet (%)^h^										
Yes	445	(47.2)	101	(42.8)	91	(39.9)	129	(54.0)	124	(51.9)
No	9	(1.0)	6	(2.5)	0	(−)	1	(0.4)	2	(0.8)
No, not required	362	(38.4)	98	(41.5)	105	(46.1)	78	(32.6)	81	(33.9)
Don’t know	40	(4.2)	8	(3.4)	11	(4.8)	8	(3.3)	13	(5.4)
Other	61	(6.5)	14	(5.9)	15	(6.6)	18	(7.5)	14	(5.9)
Change exercise habits (%)^h^										
Yes	307	(32.6)	64	(27.1)	71	(31.1)	86	(36.0)	86	(36.0)
No	119	(12.6)	42	(17.8)	30	(13.2)	25	(10.5)	22	(9.2)
No, not required	332	(35.2)	86	(36.4)	83	(36.4)	78	(32.6)	85	(35.6)
Don’t know	84	(8.9)	17	(7.2)	19	(8.3)	26	(10.9)	22	(9.2)
Other	75	(8.0)	18	(7.6)	19	(8.3)	19	(7.9)	19	(7.9)
Change alcohol consumption (%)^h^										
Yes	277	(29.4)	58	(24.6)	59	(25.9)	71	(29.7)	89	(37.2)
No	55	(5.8)	25	(10.6)	12	(5.3)	10	(4.2)	8	(3.3)
No, not required	531	(56.4)	130	(55.1)	142	(62.3)	136	(56.9)	123	(51.5)
Don’t know	30	(3.2)	9	(3.8)	4	(1.8)	10	(4.2)	7	(2.9)
Other	24	(2.5)	5	(2.1)	5	(2.2)	7	(2.9)	7	(2.9)
Willing to consider medication (% yes)^i^	241	(58.2)					112	(53.8)	129	(62.6)
Tamoxifen preference, n (%)^j^										
Pill	171	(35.7)					90	(37.7)	81	(33.9)
Cream	125	(26.2)					59	(24.7)	66	(27.6)
No preference	93	(19.5)					42	(17.6)	51	(21.3)
Neither	47	(9.8)					26	(10.9)	21	(8.8)
Don’t know	32	(6.7)					17	(7.1)	15	(6.3)
Anecdotal knowledge of tamoxifen (% yes)^j^	55	(11.8)					22	(9.4)	33	(14.1)

^a^17 women with missing value (1.8%) were excluded; ^b^ multiple answers possible; ^c^18 women with missing value (1.9%) were excluded; ^d^based on a 4-year screening interval; ^e^based on a 2-year screening interval; ^f^based on a 1-year screening interval; ^g^19 women with missing value (2.0%) were excluded; ^h^25 women with missing value (2.7%) were excluded; ^i^64 women with missing value (13.4%) were excluded; ^j^10 women with missing value (2.1%) were excluded

Only 13.1% of the women who completed the low risk scenario found a 4-year screening interval acceptable, with 27.4% of these women opting for supplemental mammography screening outside of the national screening programme. Most low-risk women (50.7%) would prefer the screening interval to remain at 2 years, and the start age to either remain at 50 years (27.9%) or increase to 55 years (16.2%). Both average-risk and moderate-risk women were presented with a 2-year screening interval, leading to more moderate-risk women perceiving a need for supplemental mammography screening than average-risk women, i.e. 50.0% versus 36.6%. Half of high-risk women found their presented 1-year screening interval acceptable (50.5%), with 43.0% of women opting for supplemental mammography screening. Almost one third of high-risk women would prefer a screening interval of 6 months and a start age of 40 years (59.4%). Of the women who would opt for supplemental mammography screening, 25.4% would not be willing to pay for the extra mammogram, 25.9% of women would pay regardless of the costs, and for 36.2% of women it would depend on the costs. Most women who completed the moderate (87.2%) or high (83.4%) risk scenarios would engage in more frequent breast self-examinations.

Women were generally in favour of changing their lifestyle to actively attempt to reduce their risk, although changing their diet was more popular than modifying alcohol intake or exercise habits, respectively. Of the women who would be willing to change their lifestyle, 31.4% would like to receive help from a dietician, whereas 24.6% would prefer to make the changes without any external help. More than half of the women who completed the moderate or high risk scenario were willing to consider taking risk-reducing medication, expressing a preference for a pill rather than a cream. Women who were not in favour of risk-reducing medication indicated that they would only like to take medication once diagnosed with breast cancer (50.9%), they did not think the benefits outweighed the harms (25.2%), or had an aversion to medication (23.9%). Most women who were in favour indicated that they felt the benefits do outweigh the harms (75.5%), but emphasised that interactions with other medications should be avoided (24.5%).

### Acceptability of risk-based breast cancer screening

Table 3 presents the results of the explorative analyses into the acceptability of risk-based screening. Assigned risk scenario was associated with increased interest in supplemental mammography (moderate vs. average OR_adj_ 1.59, 95% CI 1.09, 2.34) and increased interest in breast self-examination (moderate vs. average OR_adj_ 5.26, 95% CI 3.25, 8.52). Higher age was associated with decreased interest in knowing your breast cancer risk (OR_adj_ = 0.94, 95% CI 0.91, 0.97). Lower education was associated with increased perceived need for supplemental mammography outside of the national screening programme (higher vocational vs. lower education OR_adj_ 0.58, 95% CI 0.40, 0.84). Lower perceived breast cancer risk was associated with increased interest in breast self-examination (below average vs. average OR_adj_ 1.83, 95% CI 1.28, 2.60).

**Table 3:** Explorative analyses of factors associated with the acceptability of risk-based screening

Characteristic	Acceptability of risk-based breast cancer screening					
	Interest in risk		Interest in supplemental screening		Interest in breast self-examination	
	Unadjusted	Multi-adjusted^**c**^	Unadjusted	Multi-adjusted^**c**^	Unadjusted	Multi-adjusted^**c**^
	OR (95% CI)	OR (95% CI)	OR (95% CI)	OR (95% CI)	OR (95% CI)	OR (95% CI)
Risk scenario						
Low	n/a^d^	n/a^d^	**0.58** (0.39, 0.86)	**0.61** (0.41, 0.92)	**0.28** (0.19, 0.41)	**0.28** (0.19, 0.42)
Average	n/a^d^	n/a^d^	REF	REF	REF	REF
Moderate	n/a^d^	n/a^d^	**1.53** (1.01, 2.21)	**1.59** (1.09, 2.34)	**4.79** (3.01, 7.64)	**5.26** (3.25, 8.52)
High	n/a^d^	n/a^d^	1.15 (0.79, 1.67)	1.23 (0.84, 1.81)	**3.54** (2.29, 5.47)	**4.32** (2.72, 6.86)
Age^e^	**0.94** (0.91, 0.96)	**0.94** (0.91, 0.97)	1.00 (0.97, 1.02)	0.99 (0.97, 1.02)	1.01 (0.99, 1.03)	1.00 (0.97, 1.03)
Education						
Lower	REF	REF	REF	REF	REF	REF
Higher secondary	**1.68** (1.11, 2.55)	1.21 (0.76, 1.90)	0.79 (0.57, 1.09)	0.72 (0.51, 1.03)	1.08 (0.77, 1.53)	1.00 (0.65, 1.53)
Higher vocational	1.34 (0.89, 2.03)	1.04 (0.66, 1.61)	**0.62** (0.44, 0.88)	**0.58** (0.40, 0.84)	0.81 (0.57, 1.14)	0.66 (0.43, 1.01)
FDR^b^ breast cancer						
No	REF	REF	REF	REF	REF	REF
Yes	1.09 (0.72, 1.65)	0.97 (0.61, 1.53)	1.34 (0.98, 1.85)	1.28 (0.90, 1.81)	0.95 (0.69, 1.32)	1.00 (0.67, 1.52)
Benign breast disease						
No	REF	REF	REF	REF	REF	REF
Yes	1.20 (0.82, 1.75)	1.25 (0.83, 1.88)	1.02 (0.77, 1.37)	0.98 (0.72, 1.34)	1.03 (0.77, 1.39)	1.24 (0.86, 1.78)
General health^f^	0.99 (0.98, 1.00)	1.00 (0.98, 1.01)	1.00 (0.99, 1.01)	1.00 (0.99, 1.01)	1.01 (0.99, 1.01)	1.00 (0.99, 1.01)
Risk perception						
Below average	n/a^d^	n/a^d^	0.79 (0.60, 1.05)	0.90 (0.67, 1.22)	**1.50** (1.12, 1.99)	**1.83** (1.28, 2.60)
Average	n/a^d^	n/a^d^	REF	REF	REF	REF
Above average	n/a^d^	n/a^d^	1.75 (1.01, 3.02)	1.49 (0.82, 2.70)	1.23 (0.69, 2.17)	0.84 (0.42, 1.68)
Health anxiety^g^	1.05 (1.00, 1.09)	1.04 (0.99, 1.10)	1.03 (0.99, 1.06)	1.02 (0.99, 1.06)	1.01 (0.97, 1.04)	1.03 (0.98, 1.08)
Health locus of control						
No preference	REF	REF	REF	REF	REF	REF
Internal	1.00 (0.55, 1.80)	0.85 (0.44, 1.64)	0.84 (0.54, 1.30)	0.98 (0.61, 1.56)	1.24 (0.79, 1.95)	1.17 (0.67, 2.03)
Physician	0.56 (0.26, 1.18)	0.51 (0.22, 1.17)	0.74 (0.40, 1.37)	0.63 (0.32, 1.24)	**2.86** (1.38, 5.94)	2.09 (0.87, 5.03)
Chance	0.79 (0.45, 1.38)	0.74 (0.39, 1.39)	0.80 (0.52, 1.21)	0.87 (0.56, 1.37)	1.00 (0.65, 1.53)	0.82 (0.49, 1.39)

^a^ Odds ratios in bold are significant with *p* < 0.05; ^b^ FDR = first degree relative; ^c^ Adjusted for age, education, FDR with breast cancer, benign breast disease, general health, risk perception, risk scenario, and health anxiety; ^d^ analysis not available due to small subgroups (*n* < 10); ^e^ Age per 1 year increase; ^f^ General health per one point increase; ^g^ Health anxiety per one point increase

### Acceptability of risk-based breast cancer prevention

Table 4 presents the results of the explorative analyses into the acceptability of risk-based prevention. Assigned risk scenario was associated with willingness to change diet (high vs. average OR_adj_ 1.95, 95% CI 1.28, 2.97), willingness to limit alcohol intake (moderate vs. average OR_adj_ 2.02, 95% CI 1.33, 3.08) and willingness to consider medication (high vs. moderate OR_adj_ 1.57, 95% CI 1.03, 2.37). Higher education was associated with increased willingness to increase exercise (higher vocational vs. lower education OR_adj_ 1.93, 95% CI 1.27, 2.91) and willingness to limit alcohol intake (higher vocational vs. lower education OR_adj_ 1.81, 95% CI 1.21, 2.70). Higher BMI was associated with an increased willingness to change diet (OR_adj_ 1.12, 95% CI 1.08, 1.17) and a decreased willingness to limit alcohol intake (OR_adj_ 0.95, 95% CI 0.92, 0.98). Having at least one first-degree relative with breast cancer was associated with a decreased willingness to change diet (OR_adj_ 0.63, 95% CI 0.43, 0.93) and increase exercise (OR_adj_ 0.55, 95% CI 0.36, 0.84). Additionally, women who generally believed medicines are not overused and cause more good than harm were more interested in medication than women who did not hold these beliefs (OR_adj_ 0.87, 95% CI 0.81, 0.93; OR_adj_ 0.89, 95% CI 0.82, 0.98, respectively).

**Table 4:** Explorative analyses of factors associated with the acceptability of risk-based prevention

Characteristic	Acceptability of risk-based prevention							
	Change diet		Increase exercise		Limit alcohol intake		Consider medication	
	Unadjusted	Multi-adjusted^c^	Unadjusted	Multi-adjusted^c^	Unadjusted	Multi-adjusted^c^	Unadjusted	Multi-adjusted^c^
	OR (95% CI)	OR (95% CI)	OR (95% CI)	OR (95% CI)	OR (95% CI)	OR (95% CI)	OR (95% CI)	OR (95% CI)
Risk scenario								
Low	1.12 (0.76, 1.66)	1.10 (0.73, 1.68)	0.80 (0.52, 1.21)	0.76 (0.49, 1.18)	0.98 (0.64, 1.50)	1.08 (0.69, 1.68)	n/a	n/a
Average	REF	REF	REF	REF	REF	REF	n/a	n/a
Moderate	**1.88** (1.27, 2.80)	**1.95** (1.27, 2.98)	1.33 (0.88, 2.01)	1.30 (0.84, 2.00)	1.27 (0.84, 1.92)	1.32 (0.86, 2.04)	REF	REF
High	**1.72** (1.16, 2.56)	**1.95** (1.28, 2.97)	1.28 (0.85, 1.93)	1.37 (0.89, 2.09)	**1.77** (1.19, 2.65)	**2.02** (1.33, 3.08)	1.44 (0.97, 2.13)	**1.57** (1.03, 2.37)
Age^f^	**0.98** (0.96, 1.00)	0.99 (0.96, 1.01)	0.98 (0.96, 1.00)	0.99 (0.96, 1.01)	1.01 (0.98, 1.03)	1.00 (0.98, 1.03)	0.98 (0.95, 1.01)	0.99^d^ (0.96, 1.03)
Education								
Lower	REF	REF	REF	REF	REF	REF	REF	REF
Higher secondary	1.21 (0.86, 1.72)	1.16 (0.79, 1.71)	**1.86** (1.27, 2.73)	**1.81** (1.20, 2.74)	1.41 (0.97, 2.06)	1.35 (0.90, 2.03)	1.33 (0.80, 2.19)	1.26 (0.73, 2.16)
Higher vocational	0.97 (0.68, 1.38)	1.06 (0.71, 1.56)	**1.93** (1.31, 2.85)	**1.93** (1.27, 2.91)	**2.03** (1.38, 2.97)	**1.81** (1.21, 2.70)	1.38 (0.82, 2.32)	1.56 (0.89, 2.74)
BMI^g^	**1.13** (1.09, 1.17)	**1.12** (1.08, 1.17)	1.02 (0.99, 1.05)	1.02 (0.98, 1.05)	**0.94** (0.91, 0.97)	**0.95** (0.92, 0.98)	1.04 (1.00, 1.08)	1.03 (0.99, 1.08)
FDR^b^ breast cancer								
No	REF	REF	REF	REF	REF	REF	REF	REF
Yes	0.72 (0.51, 1.00)	**0.63** (0.43, 0.93)	**0.58** (0.40, 0.85)	**0.55** (0.36, 0.84)	0.84 (0.59, 1.20)	0.90 (0.61, 1.33)	1.53 (0.94, 2.47)	1.31 (0.76, 2.25)
Benign breast disease								
No	REF	REF	REF	REF	REF	REF	REF	REF
Yes	**0.72** (0.53, 0.98)	0.88 (0.63, 1.23)	0.83 (0.60, 1.14)	0.86 (0.61, 1.22)	1.06 (0.77, 1.44)	0.99 (0.71, 1.38)	1.16 (0.74, 1.83)	1.26 (0.78, 2.04)
Co-morbidity								
0–1	REF	REF	REF	REF	REF	REF	REF	REF
≥ 2	1.13 (0.86, 1.49)	1.00 (0.73, 1.38)	1.14 (0.85, 1.53)	1.20 (0.87, 1.66)	0.79 (0.59, 1.05)	0.90 (0.65, 1.24)	1.23 (0.83, 1.82)	1.06 (0.69, 1.65)
General health^h^	**0.99** (0.98, 1.00)	0.99 (0.98, 1.01)	**0.99** (0.98, 1.00)	**0.99** (0.98, 1.00)	1.00 (0.99, 1.01)	1.00 (0.99, 1.01)	**0.98** (0.97, 1.00)	0.99^d^ (0.97, 1.01)
Life events								
0–1	REF	REF	REF	REF	REF	REF	REF	REF
≥ 2	1.15 (0.84, 1.56)	1.08 (0.78, 1.51)	1.01 (0.72, 1.40)	1.00 (0.70, 1.41)	0.92 (0.67, 1.26)	0.93 (0.66, 1.30)	1.06 (0.68, 1.66)	1.05 (0.65, 1.69)
Risk perception								
Below average	0.86 (0.65, 1.15)	0.92 (0.67, 1.26)	0.96 (0.71, 1.30)	0.97 (0.70, 1.34)	1.16 (0.86, 1.56)	1.04 (0.75, 1.43)	**0.56** (0.37, 0.85)	0.65 (0.41, 1.01)
Average	REF	REF	REF	REF	REF	REF	REF	REF
Above average	**1.96** (1.04, 3.68)	1.72 (0.85, 3.50)	1.23 (0.67, 2.27)	1.40 (0.71, 2.77)	1.04 (0.56, 1.90)	0.88 (0.45, 1.72)	1.21 (0.54, 2.69)	1.00 (0.41, 2.46)
Health anxiety^i^	**1.05** (1.02, 1.09)	1.04 (1.00, 1.08)	1.03 (0.99, 1.06)	1.01 (0.97, 1.05)	1.02 (0.98, 1.06)	1.03 (0.99, 1.07)	1.04 (0.99, 1.09)	1.02 (0.96, 1.08)
Health locus of control								
No preference	REF	REF	REF	REF	REF	REF	REF	REF
Internal	0.96 (0.60, 1.51)	0.94 (0.57, 1.55)	1.13 (0.70, 1.82)	1.05 (0.63, 1.75)	1.47 (0.91, 2.39)	1.50 (0.89, 2.53)	1.01 (0.50, 2.08)	0.98 (0.46, 2.09)
Physician	**2.25** (1.12, 4.50)	1.77 (0.82, 3.80)	2.23 (1.14, 4.36)	2.05 (0.99, 4.25)	1.24 (0.62, 2.46)	1.44 (0.68, 3.03)	1.23 (0.50, 3.04)	1.23 (0.46, 3.29)
Chance	0.93 (0.60, 1.44)	1.01 (0.63, 1.64)	1.14 (0.72, 1.81)	1.19 (0.73, 1.95)	1.20 (0.75, 1.92)	1.27 (0.76, 2.10)	0.75 (0.38, 1.49)	0.66 (0.31, 1.38)
Current medication use								
No							REF	REF
Yes							1.33 (0.89, 1.98)	1.00^e^ (0.63, 1.59)
Beliefs about medicines^j^								
Harm							**0.87** (0.80, 0.94)	**0.89**^**d**^ (0.82, 0.98)
Overuse							**0.86** (0.81, 0.92)	**0.87**^**d**^ (0.81, 0.93)

^a^ Odds ratios in bold are significant with p < 0.05; ^b^FDR = first degree relative; ^c^ Adjusted for age, education, BMI, FDR with breast cancer, benign breast disease, general health, risk perception, risk scenario, and health anxiety; ^d^ Additionally adjusted for current medication use; ^e^ Additionally adjusted for beliefs about medicines; ^f^ Age per 1 year increase; ^g^ BMI per one point increase; ^h^ General health per one point increase; ^I^ Health anxiety per one point increase; ^j^ Beliefs about medicines per one point increase

## Discussion

Our findings show that Dutch women are generally interested in their breast cancer risk and open to tailored screening and prevention strategies. However, there are some important considerations that need to be addressed to facilitate potential future implementation.

We found that around 80% of women wanted to know their breast cancer risk. This is in line with previously reported estimates which vary between 74% and 95% [5, 6, 16]. It shows promise regarding women’s willingness to engage with breast cancer risk information. Moreover, most women did not object to providing the information required to assess breast cancer risk, i.e. a mammogram, blood sample, and questionnaire data. We found that 10% of women were unsure about whether they would want to know their breast cancer risk. Our previous focus group study indicated several underlying factors affecting women’s indecision, such as perceived emotional burden and lack of self-efficacy [8]. It additionally showed that women who were unsure about participation in the new risk-based screening programme tended to perceive a greater need for information [8]. This underlines the importance of comprehensive information materials which will enable women to make an informed decision regarding participation.

Dutch women saw merit in risk-based screening strategies. In line with previous research, annual screening was preferred by women completing the high risk scenario and around half of the women who completed the low risk scenario preferred to maintain the current screening interval of 2 years [16]. Concerns about women’s reluctance to decrease screening intensity have been reported before [7, 16, 17]. Women have previously questioned the scientific basis of less intensive screening, perceiving it as service rationing rather than good evidence-based practice [7, 17]. A fear of missed cancers was another major barrier to uptake of less intensive screening [16, 18]. However, our results show that some hypothetical low-risk women would accept a longer screening interval, with one third indicating a preference for a screening interval of 3–5 years. This corresponds to findings from a study performed in the United Kingdom, which showed that around 50% of women would consider a screening interval of 4 or 5 years if low risk [16]. Moreover, 37% of women would accept no screening if very low risk [16]. Most women who completed the low risk scenario (73.3%) did not perceive a need for supplemental mammography screening outside of the national programme when presented with a hypothetical screening interval of 4 years. This suggests that women are open to less intensive screening if this optimises the benefit-to-harm ratio of screening for low-risk women by reducing, e.g., the number of false positives. It provides an opportunity to offset the costs associated with mass risk assessment and more intensive screening for high-risk women.

Women’s preferences regarding the organisation of a potential risk-based screening and prevention programme will likely stretch healthcare resources. With around 90% of women who completed the moderate or high risk scenario wanting a risk consultation from a GP or oncologist, the involvement of other healthcare professionals in the screening process will increase. In many European countries, GPs are an obvious choice due to their accessibility and knowledge of a woman’s personal (medical) history. However, which professional is most suitable will depend on how healthcare is funded and arranged in a country. Previous research has shown that (primary) healthcare professionals generally lack knowledge on breast cancer risk communication and the latest screening and prevention guidelines, making additional training unavoidable [19, 20]. In addition, more than 40% of women with a hypothetical average, moderate or high breast cancer risk perceived a need for supplemental mammography screening, based on their assigned screening interval of two, two, and 1 year, respectively. This is particularly conspicuous in the case of average risk women for whom the recommended screening strategy corresponded to current practice in the Netherlands. It indicates that risk communication could make women more anxious and breast cancer aware. The overall psychological impact of receiving breast cancer risk information in a screening setting appears negligible [6]. However, considerable variation in understanding of risk has been demonstrated, potentially hindering informed decision-making. Perceived need for supplemental mammography screening was also associated with a lower education. Since this is a relatively new field of research, there is insufficient scientific literature available to be able to compare all our explorative results. This finding does, however, emphasise the importance of knowledge and comprehensive information leaflets. We need to be meticulous in our information provision about the interpretation of a woman’s breast cancer risk, and the advantages and disadvantages of the proposed screening and prevention strategies.

The potential of increased breast cancer awareness after risk communication was affirmed by women’s perceived need for increased breast self-examination when completing the moderate or high risk scenario. Women have previously reported that breast self-examinations are not part of their routine, yet being aware of their risk would make them feel more inclined to engage in the practice [8]. However, women emphasised that they feel insufficiently capable of performing correct breast self-examinations, expressing a need for more education [8]. Although it may help women feel more empowered, increasing their perceived sense of control over identifying the disease, research has shown that it has no clear benefits in addition to breast cancer screening [21, 22]. However, breast self-examination can make women more familiar with their breasts, facilitating awareness of any potential changes. Notably, we found that women’s own perceived breast cancer risk was associated with a decreased need for breast self-examination. Women reported their own perceived breast cancer risk before they were randomly assigned to a hypothetical risk and subsequent screening scenario. The discordant finding indicates that women were able to follow the instruction to disengage from their own perceived breast cancer risk, and instead empathise with the assigned risk scenario. It remains unclear whether women would actually want to engage in breast self-examination more after risk feedback or whether they provided socially desirable answers in the context of the risk scenario.

Although we previously found that women’s acceptability of preventative measures for breast cancer was limited [8], this survey provides a different picture. The majority of women who felt they would benefit from lifestyle changes were willing to change their diet, increase their level of exercise, and decrease their alcohol intake. Higher assigned breast cancer risk and higher BMI resulted in increased willingness to change dietary habits, indicating positive interest from those women who may benefit most. Around one third of our survey participants would like to receive help from a dietician, with significantly more women with a higher BMI preferring professional help. This is in accordance with results from the PROCAS lifestyle study, which offered two weight control programmes to overweight or obese women in the UK National Health Service Breast Screening Programme (NHSBSP) identified at low, average, moderately increase or high-risk of breast cancer [23]. They found that women who were informed to be at increased risk of breast cancer were more likely to join and remain in the programmes. These women consequently lost more weight than women who were not at increased risk [23]. Women with a first degree family history of breast cancer appear less interested in changing their dietary and exercise habits. Women have previously reported a sceptical attitude towards the scientific link between lifestyle and breast cancer risk due to mixed messages in the media [8]. Particularly those who were at increased risk of breast cancer due to a genetic predisposition questioned the effect that lifestyle changes could have on their breast cancer risk [8]. Adversely, higher education appears to increase interest in preventative lifestyle changes. Therefore, comprehensive evidence-based information materials which unequivocally present the latest research on lifestyle and breast cancer may assist in informing and motivating women.

Although low uptake of risk-reducing medication for breast cancer has been widely reported [24], our results show that more than half of Dutch women who completed the moderate and high risk scenarios were willing to consider medication. A main barrier was perceived level of side effects. Currently the same dose of tamoxifen is prescribed for preventative and adjuvant use. However, the Karma Intervention Study (Karisma - https://karmastudy.org/ongoing-research) is investigating the minimal effective dose of tamoxifen, monitoring the accompanying side effects of each dose. Lowering the required dose of tamoxifen without compromising its risk-reducing effects may make the medication more accessible and acceptable to eligible women. Additionally, a considerable number of women professed a preference for topical tamoxifen, making further research into its effectiveness compared with oral tamoxifen worthwhile [25].

### Strengths and limitations

This is the first study to comprehensively explore women’s perceptions of an integrated risk-based breast cancer screening and prevention programme. However, some caution in the interpretation of our results is warranted. Firstly, because risk-based breast cancer screening has not yet been implemented and comprehensive data on women’s breast cancer risk is not yet routinely collected, we had to rely on hypothetical risk scenarios and proxy outcome measures. Although it appears that women were able to reason within their assigned risk category, the stakes are inherently lower, and it is unclear how this may have affected the results. Although women expressed positive intent regarding participation in risk-based screening and prevention strategies, previous research has shown that the role of intent is limited in this context [26]. Therefore, it remains unclear how women’s intent will actually translate to uptake and adherence if risk-based breast cancer screening and prevention is implemented.

Moreover, our findings are based on a selection of women who have previously participated in both breast cancer screening and the PRISMA study. This could account for the relatively large number of women with a first-degree family history of breast cancer (21.7%) in our study sample. Previous studies performed in the Dutch breast cancer screening population found that around 4.5–5.6% of women reported a first-degree family history of breast cancer [27, 28]. Additionally, we had a relatively low response rate (18.4%). We were able to compare survey participants to survey invitees who had previously completed the PRISMA study questionnaire (49.61%) on key characteristics (Supplement 1), which presented no considerable differences. However, it is still imaginable that the women who participated in this survey study are both more breast cancer aware and more invested in breast cancer early detection and prevention. It is therefore unclear whether the perceptions of our participants are directly generalizable to those of women who did not participate in the PRISMA study or currently do not participate in breast cancer screening. Future research is required to confirm our findings in a more diverse group of women.

## Conclusions

Dutch women generally appear in favour of receiving their breast cancer risk estimate with subsequent tailored screening and prevention recommendations. However, educating women on the benefits and harms of all risk-based screening and prevention strategies is key to acceptability and informed decision-making. Women’s preferences for preventative strategies in particular are diverse. Therefore, we need to engage women in decisions about tailored preventative strategies to optimise uptake and adherence.

## Supplementary information

**Additional file 1 **: **Supplement 1.** Key characteristics of all survey participants and the proportion of invitees who previously completed the PRISMA study questionnaire

## Data Availability

The datasets used and analysed during the current study are available from the corresponding author on reasonable request.

## Abbreviations

BMI: Body mass index
FDR: First degree relative
HLoC: Health locus of control
MHT: Menopausal hormone therapy
PRISMA: Personalised RISk-based MAmmography screening
PROCAS: Predicting Risk Of breast CAncer at Screening
