# Supplementary material for: Dutch women’s intended participation in a risk-based breast cancer screening and prevention programme: a survey study identifying preferences, facilitators and barriers
Source: BMC Cancer. 2020 Oct 6;20:965. doi: 10.1186/s12885-020-07464-2 (PMC7539478; doi:10.1186/s12885-020-07464-2)
Supplement: Supplementary file 1 — Additional file 1 : Supplement 1. Key characteristics of all survey participants and the proportion of invitees who previously completed the PRISMA study questionnaire [file 12885_2020_7464_MOESM1_ESM.docx]

**Supplement 1.** Key characteristics of all survey participants and the proportion of invitees who previously completed the PRISMA study questionnaire

|  | **Participants**  **N=942** | | **Invitees***  **N=2,535** | |
| --- | --- | --- | --- | --- |
| Age (years), mean (SD) | 59.0 | (6.3) | 59.7 | (6.7) |
| Education level, n (%) |  |  |  |  |
| Lower education | 249 | (26.4) | 779 | (30.8) |
| Higher secondary education | 372 | (39.5) | 974 | (38.5) |
| Higher vocational qualification | 321 | (34.1) | 778 | (30.7) |
| First degree family history breast cancer, n yes (%) | 204 | (21.7) | 484 | (19.1) |
| Benign breast disease, n yes (%) | 272 | (28.9) | 713 | (28.1) |

* We invited 5,110 women who had previously participated in the PRISMA study to participate in this follow-up study; of these women 2,535 (49.61%) had previously completed the PRISMA study questionnaire. We were unable to distinguish participants from non-participants due to the anonymous nature of the follow-up survey.
